# Immediate Effects of Stabilization Exercises on Trunk Muscle Activity during Jump Header Shooting: A Pilot Study

**DOI:** 10.3390/healthcare10071272

**Published:** 2022-07-09

**Authors:** Chie Sekine, Kazusa Saisu, Ryo Hirabayashi, Hirotake Yokota, Haruna Hayashi, Tomoya Takabayashi, Mutsuaki Edama

**Affiliations:** 1 Department of Physical Therapy, Niigata University of Health and Welfare, 1398 Shimami-cho, Kita-ku, Niigata City 950-3198, Japan; sekine@nuhw.ac.jp (C.S.); rpa18056@nuhw.ac.jp (K.S.); hirabayashi@nuhw.ac.jp (R.H.); yokota@nuhw.ac.jp (H.Y.); rpa18104@nuhw.ac.jp (H.H.); takabayashi@nuhw.ac.jp (T.T.); 2 Institute for Human Movement and Medical Sciences, Niigata University of Health and Welfare, 1398 Shimami-cho, Kita-ku, Niigata City 950-3198, Japan

**Keywords:** jump header shooting, soccer, trunk muscle activity, stabilization exercise

## Abstract

This study aimed to clarify trunk muscle activity during jump header shooting and examine the immediate effects of trunk stabilization exercises on trunk muscle activity. Nineteen males who had played soccer for over 5 years were assigned to either the trunk stabilization exercise group or the control group. Muscle activity during jump header shooting was measured before and after intervention. The intervention in the trunk stabilization exercise group was trunk muscle training, whereas that in the control group was sitting. The phases of jump header shooting and the effects of the interventions were compared. In pre-intervention measurements, the internal oblique activity during the push-off phase and early floating phase was significantly greater than that during the late floating phase (*p* < 0.01667). In pre-intervention measurements, the muscle activity of the internal oblique increased from the push-off phase, prior to the increase in muscle activity of the rectus abdominis and external oblique, whereas the muscle activity of all abdominal muscles increased immediately after take-off. The trunk stabilization exercise intervention decreased the muscle activity of the erector spinae (*p* < 0.05). There seems to be a certain activation sequence in the abdominals during jump header shooting, and a single application of stabilization exercises could possibly reduce the activation of the back muscles.

## 1. Introduction

Heading has emerged as an important skill in defensive and offensive football [1]. Headers can be decisive in gaining or losing ball possession and are often critical to the game outcome [2]. Therefore, soccer players must be able to head the ball with a high-level skill.

During heading, players are instructed by their coaches to tuck the chin and tighten the abdominal muscles upon the ball’s contact with the head. Moreover, the importance of trained and stable neck and trunk muscles for headers is increasingly being discussed [3]. Understanding trunk muscle activity during heading is important for instructing and coaching players on various aspects of heading; however, it remains unexplored yet, and the abdominal and back muscles activity is not clear.

Jump header shooting includes take-off, floating in midair, and landing. Muscle activity of the abdominal and back muscles has been investigated during jumping and landing, which includes take-off, floating in midair, and landing, all of which are part of jump header shooting. The previous studies reported that intra-abdominal pressure and muscle activity of the abdominal muscles increased immediately prior to foot contact of the landing [4], and the rectus abdominis (RA) and external oblique (EO) muscles were highly activated [5]. There is co-contraction of all abdominal muscles during the push-off phase, which is influenced by the ground reaction force during the standing long jump [6]. Thus, trunk muscle activity and function are considered important for jumping and landing [4,5,6]. Therefore, trunk muscle activity including abdominal and back muscles may be important for jump header shooting.

The ball impact is characteristic of heading. Muscle activity during ball impact has been reported in heading and soccer kick [3,7,8]. Regarding heading, neck muscle activity has been investigated [3,7]. A study reported that the neck muscles act to dissipate the force of impact when the ball contacts the head and to stabilize the connection between the head and body [7]. Similarly, during a soccer kick, the activity of the hip adductor muscles increases before ball impact [8]. The increased activity of the adductor and neck muscles is thought to stabilize the trunk prior to ball impact. During the ball impact of heading, the soccer player is instructed that it is important to stabilize the trunk, including the neck. However, the abdominal and back muscle activity during heading is not clear. Therefore, investigating the trunk muscle activity during jump header shooting is imperative to provide basic data for coaching.

Trunk stabilization exercise training has been shown to improve trunk muscle function. Recent systematic reviews have presented some evidence that stabilization exercises improve jump performance [9,10]. For example, the jump height of adolescent soccer players improved after a 6-month trunk muscle training intervention [11]. A study that examined the immediate effects of conventional trunk exercises and trunk stabilization exercises reported that rebound jump performance improved in only the stabilization exercise group [12]. These results suggest that trunk stabilization exercises may have immediate effects on jump header shooting, although they have not been investigated yet. Trunk stabilization exercises may improve lumbar segmental stabilization [13] and decrease erector spinae (ES) muscle activity.

This study aimed to clarify trunk muscle activity during jump header shooting and examine the immediate effects of trunk stabilization exercises on trunk muscle activity. We hypothesized that the abdominal muscles under investigation would be highly activated before ball contact prior to the intervention and that the muscle activity of the abdominal and the erector spinae muscles would be altered even after a single trunk stabilization exercise intervention session.

## 2. Materials and Methods

### 2.1. Participants

Nineteen male college students (age: 20.7 ± 1.1 years, height: 173.1 ± 5.1 cm, weight: 65.6 ± 7.5 kg, soccer experience: 10.3 ± 2.3 years) who had played soccer in junior high and high school clubs and youth sports teams for over 5 years were recruited. Each participant was assigned to either the trunk stabilization exercise group (*n* = 10) or the control group (*n* = 9) randomly. The exclusion criterion was a history of lower extremity surgery. None of the participants suffered orthopedic injuries or pain that might have impeded their performance. The participants were informed of the study’s aim and procedures before participation, and written informed consent was obtained from all participants. The study was conducted according to the tenets of the Declaration of Helsinki and was approved by the institutional ethics review committee (Approval number: 18579-210218). This study was registered in the UMIN Clinical Trials Registry (UMIN000047651).

### 2.2. Experimental Task

**Figure 1:**
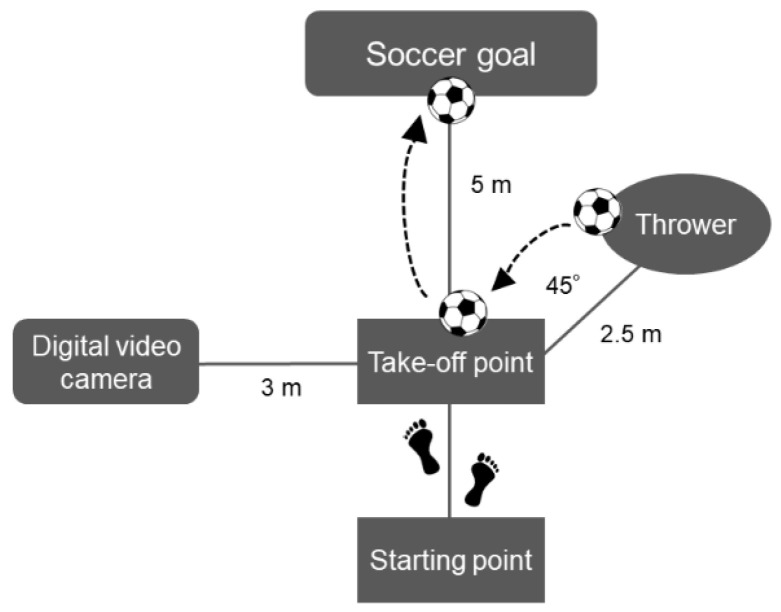
Experimental task.

The experimental task involved jump header shooting. The approach was standardized in two steps. The first step involved the right leg, and the second step involved the left leg. The participants took off on the second step, and the take-off position was 5 m ahead of the goal post (Figure 1). Take-off and landing were either on the left leg only or both legs with the left leg as the axis. A thrower threw the soccer ball (MC5-WBL; MIKASA Co., Ltd., Hiroshima, Japan) 2.5 m from the take-off point and at a 45° right angle, at the take-off point. The target height of the soccer ball was set at 1.2 times the participant’s height at the take-off point. The height from the floor was measured using a measuring tape (Steel Handy Measure SMS-2012; KONYO Co. Ltd., Niigata, Japan), and the target height was defined. The same thrower threw the ball in all the trials. If the following three conditions were met, the trial was considered to be cleared: (1) participants scored a goal, (2) the target height of the soccer ball was in accordance with the rules, and (3) the participants’ movements were in accordance with the rules. A jump header shooting trial was performed before and after intervention until three cleared trials were obtained. The parameters in three cleared trials were averaged and analyzed. The experimental task applied in this study was based on previous studies [3,7], and the procedure was determined after multiple preliminary experiments with a coauthor with extensive experience in jump header shooting.

The experimental task was standardized for all trials. Participants shot to the goal post 5 m ahead of the take-off point.

### 2.3. Data Measurement

Muscle activity was measured using a wireless surface electromyography (EMG) system (DELSYS Trigno Wireless EMG System; DELSYS, Natick, MA, USA). The sampling frequency was 2000 Hz. The activity of the left-sided muscles was measured. Before applying the electrodes, shaving and abrading to prep the skin were performed. The skin was rubbed with alcohol to reduce skin impedance, and surface electrodes were positioned on the left RA (3 cm lateral to the umbilicus), IO (1 cm medial and inferior to the anterior superior iliac spine), EO (15 cm lateral to the umbilicus), ES (3 cm lateral to the L3 spinous process), gluteus maximus (GMa, midway between the greater trochanter and sacrum), rectus femoris (RF, the point corresponding to 50% of the distance between the ASIS and upper margin of the patella), biceps femoris (BF, the point corresponding to 50% of the distance between the head of the fibula and the ischial tuberosity), and gastrocnemius medial head (MG, one handbreadth below the popliteal crease on the medial mass of the calf) based on previous studies [14,15,16]. The surface electrodes were attached parallel to the muscle fibers.

A digital video camera (Exilim EX-FH20; Casio Computer Co., Ltd., Tokyo, Japan) was set up perpendicular to the plane of motion, 3 m to the left of the take-off point; the recordings were made at a frequency of 210 Hz. To obtain kinematic data, reflective markers (QPM190, Qualisys AB, Gothenburg, Sweden) were attached to the left lateral epicondyle of the femur. The digital video camera was synchronized with an electromyogram system.

### 2.4. Experimental Procedure

**Figure 2:**
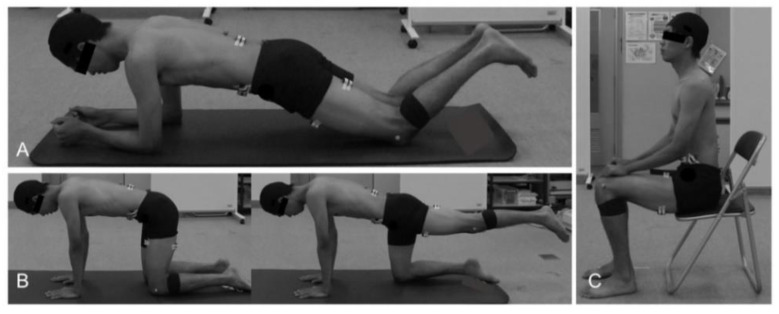
Trunk stabilization exercises (**A**,**B**) and chair sitting position (**C**). (**A**) Elbow-knee position, (**B**) hand-knee position with left lower extremity extension, and (**C**) chair sitting position.

For normalization of EMG data, the participants practiced maximum voluntary contraction (MVC) to learn the position and measurement method before the MVC test. The MVC test was conducted only once because it led to fatigue in the participant, which might have affected the jump header shooting. Manual resistance was applied until the maximum effort was reached, and the participants performed maximum isometric contractions for 3 s. After the muscle activity during MVC was recorded, the thrower practiced throwing the ball to the target point, and participants then practiced the jump header shooting. When practicing throwing the ball, a platform was placed at the take-off point, and one person stood on the platform to catch the ball. After the shooting practice, reflective markers were attached to the left lateral epicondyle of the femur, and pre-intervention measurements were taken. The intervention in the trunk stabilization exercise group was trunk muscle training, whereas that in the control group was sitting (Figure 2). Two different trunk stabilization exercises were performed, with 1-minute rest between exercises. The same physical therapist with clinical experience of over 5 years coached the participants on all exercises. The participants were instructed to maintain a neutral position of the spine during the exercise. First, the participant was instructed to maintain the elbow-knee position [17], followed by the prone plank position on the floor for 30 s, such that the elbows were beneath the shoulders and the upper arms were perpendicular to the floor. Second, the participant was instructed to assume a hand-knee position [18] and to perform left lower extremity extension exercises. Using a metronome (ME110SBL; Yamaha Corporation, Shizuoka, Japan), the participants extended their left lower extremity for 2 s, held it in an extension position for 2 s, returned to the starting posture in the next 2 s, and maintained the starting posture for 2 s. The duration of one set of this exercise was 8 s, and 10 sets were conducted consecutively. The control group maintained the chair sitting position for 170 s, which is the time required for trunk stabilization exercises. Post-intervention measurements were started within 3 min after intervention.

### 2.5. Data Analysis

**Figure 3:**
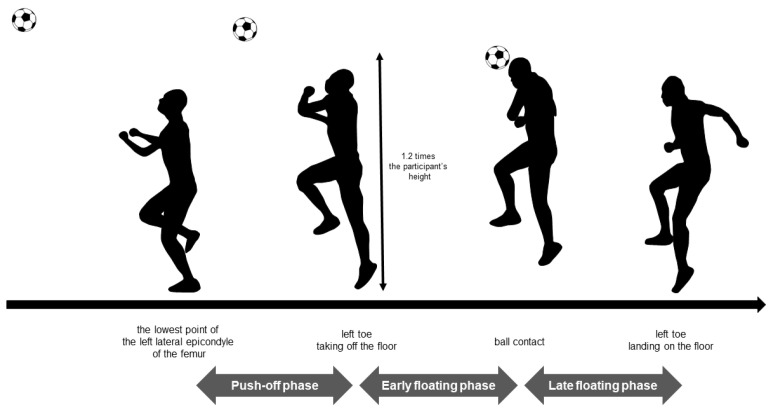
Phases of the jump header shooting. The target height of the soccer ball was set at 1.2 times the participant’s height at the take-off point.

A data analysis software (ImageJ; National Institutes of Health, Bethesda, MD, USA) was used to divide jump header shooting into the following three phases: the push-off phase, early floating phase, and late floating phase (Figure 3). The time between the lowest point of the left lateral epicondyle of the femur and the take-off on the left toe was defined as the push-off phase. The time between the take-off on the left toe and the ball contact was defined as the early floating phase. The time between the ball contact and the left toe landing on the floor was defined as the late floating phase. The raw data were bandpass-filtered between 20 and 450 Hz and full-wave rectified using the analysis software (LabChart 8; AD Instruments, Dunedin, New Zealand). The root-mean-square (RMS) during the MVC test was calculated by identifying the compartment with the maximum amplitude of 0.1 s. The RMS during each phase was normalized as a percentage of the greatest RMS obtained during a 0.1 s period in the MVC test (%MVC). The hang time, which is the time from the take-off on the left toe to the left toe landing on the floor, was calculated.

### 2.6. Statistical Analysis

Height, weight, age, and soccer experience were compared between the trunk stabilization exercise group and the control group. To confirm the normality of the data, we conducted the Kolmogorov–Smirnov test. A nonparametric test was selected for age. All participants were included in the analysis, and the muscle activities of the pre-intervention measurements were compared. To confirm the normality of the data, we conducted the Kolmogorov–Smirnov test. As a result of the normality test, nonparametric test was selected for IO, EO, GMa, RF, and BF since these muscles do not meet the assumption of normality. As a result of the normality test, a one-way repeated measure analysis of variance (push-off, early floating, and late floating) was performed to compare the pre-intervention muscle activity measurements of the RA, ES, and MG between the phases. The Bonferroni correction was performed as a post hoc test. Friedman’s test was performed to compare the pre-intervention muscle activity measurements of the IO, EO, GMa, RF, and BF between the phases. If there was a significant difference, Wilcoxon signed-rank tests were performed.

The effects of trunk stabilization exercises were also analyzed. Two-way repeated-measures analysis of variance of the measurements (pre-intervention and post-intervention) and phases (push-off, early floating, and late floating) was used to compare all muscle activities in the trunk stabilization exercise group. Similarly, a two-way repeated-measures analysis was conducted in the control group. The Bonferroni correction was performed as a post hoc test. Paired t-tests were conducted between the two measurements (pre-intervention vs. post-intervention) for each hang time.

Statistical analyses were performed using SPSS version 28.0 (IBM Corp., Armonk, NY, USA). The nominal scale for each paired comparison using Wilcoxon signed-rank tests was set at 0.01667, which was calculated by dividing the 0.05 significance level by the total number of paired comparisons. The statistical significance of the other tests was set at a *p*-value of 0.05. *p*-values were corrected for multiple comparisons using the Bonferroni correction method by multiplying *p*-values by the number of comparisons. Adjusted *p*-values less than 0.05 were regarded as significant.

## 3. Results

**Figure 4:**
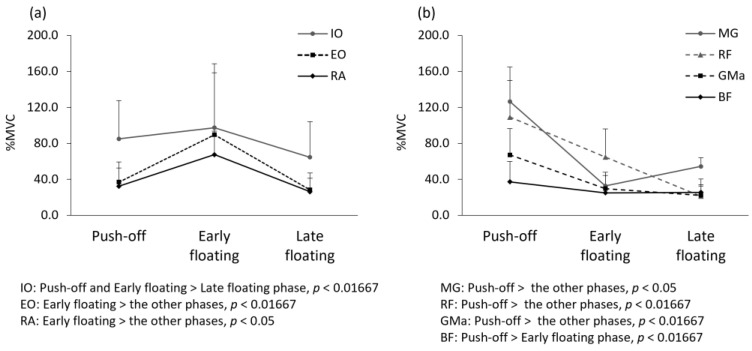
Comparison of muscle activity by the jump header shooting phases in pre-intervention. Mean and standard deviation for the electromyographic activity of (**a**) abdominal muscles and (**b**) lower extremity muscles in pre-intervention. Abbreviations: IO—internal oblique; EO—external oblique; RA—rectus abdominis; MG—gastrocnemius medial head; RF—rectus femoris; GMa—gluteus maxims; BF—biceps femoris; MVC—maximum voluntary contraction.

There were no significant differences in height, weight, and soccer experience between the groups, but age was significantly different (Table 1). The average number of pre-intervention trials was 5.0 ± 1.7 times, and that of post-intervention trials was 4.7 ± 1.3 times. All EMG data are expressed as %MVC. For the pre-intervention measurements, the muscle activity of the RA and EO during the early floating phase was significantly greater than that during the other phases (RA: *p* < 0.05, partial *η*^2^ = 0.82; EO: *p* < 0.01667, r = 0.88; Figure 4). The muscle activity of the IO during the push-off phase and early floating phase was significantly greater than that during the late floating phase (*p* < 0.01667, push-off vs. late floating: r = 0.6, early vs. late floating: r = 0.76; Figure 4). The muscle activity of the GMa, RF, and MG during the push-off phase was significantly greater than that during the other phases (GMa: *p* < 0.01667, push-off vs. early floating: r = 0.84, push-off vs. late floating: r = 0.88; RF: *p* < 0.01667, r = 0.88; MG: *p* < 0.05, partial *η*^2^ = 0.906; Figure 4). The muscle activity of the BF during the push-off phase was significantly greater than that during the early floating phase (*p* < 0.01667, r = 0.64; Figure 4).

**Figure 5:**
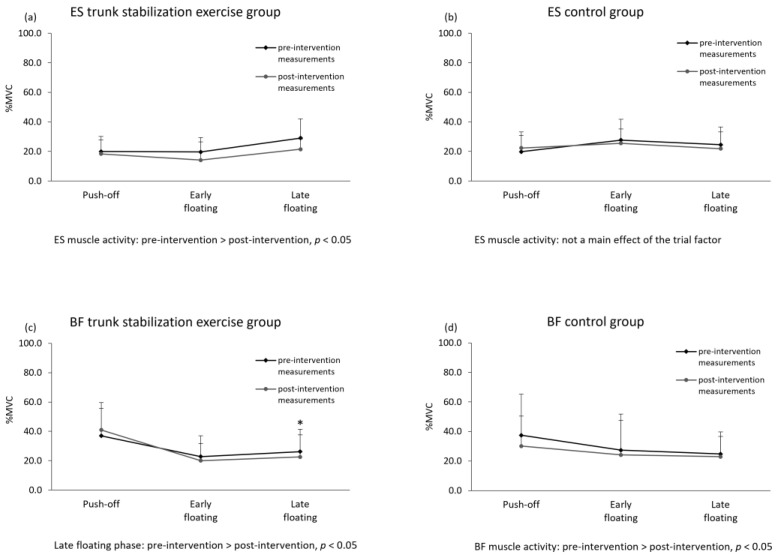
Comparison of pre- and post-intervention muscle activities. Abbreviations: ES—erector spinae; BF—biceps femoris; MVC—maximum voluntary contraction.

In the trunk stabilization exercise group, ES muscle activity showed a main effect of phase and trial; it was significantly decreased at post-intervention compared with that at pre-intervention (*p* < 0.05, partial *η*^2^ = 0.453; Table 2, Figure 5). Significant interactions between the measurements and phases in the BF were found in the trunk stabilization exercise group. The post hoc test results demonstrated that the BF muscle activity in the late floating phase was significantly decreased at post-intervention compared with that at pre-intervention (*p* < 0.05, partial *η*^2^ = 0.478; Table 2, Figure 5). In the control group, BF muscle activity showed a main effect of the trial; it was significantly decreased at post-intervention compared with that at pre-intervention (*p* < 0.05, partial *η*^2^ = 0.535; Table 2, Figure 5). There was no significant difference in the pre-intervention and post-intervention hang times in both groups (exercise group: *p* = 0.625, d = 0.78, control group: *p* = 0.364, d = 0.983; Table 3).

## 4. Discussion

This study aimed to clarify trunk muscle activity during jump header shooting and to examine the immediate effects of trunk stabilization exercises on trunk muscle activity. In the assessment of muscle activity for all subjects (*n* = 19), the hypothesis was that the abdominal muscles would be highly active before ball contact. In pre-intervention and post-intervention comparison, it was hypothesized that muscle activity in the left erector spinae muscle decreases after trunk stabilization exercise intervention. The results of this study supported both our hypotheses. With respect to pre-intervention measurements, the muscle activity of the RA and EO during the early floating phase was significantly greater than that during the other phases. In addition, the muscle activity of the IO during the push-off phase and early floating phase was significantly greater than that during the late floating phase. Therefore, it is suggested the muscle activity of the IO increases from the push-off phase prior to the increase in muscle activity of the RA and EO, and the muscle activity of all abdominal muscles increases immediately after take-off. Previous studies have reported that the activity of the abdominal muscles increases during the landing phase of a jump [4,5]. In addition, as for trunk muscle activity during standing long jump, abdominal muscle activity is the greatest during the push-off phase and lower after take-off [6]. Thus, the abdominal muscles are most active during take-off and landing. In contrast, in the present study, the muscle activity of the IO increased during the push-off phase, and the muscle activity of all abdominal muscles increased immediately after take-off. The cervical muscles are included in the trunk and stabilize the head before ball contact [7]; these muscle activities can reduce the impact of ball contact [19]. In this study, similar to the cervical muscles, abdominal muscle activity may have been greater in the early floating phase in order to improve trunk stability in preparation for ball contact. The transversus abdominis (deep trunk muscle) is activated earlier than the RA and EO in the standing long jump [6]. In this study, the IO, which also acts as a deep muscle of the trunk, showed greater activity from take-off before that of the RA and EO. These results are similar to those of a previous study [6]. The results of the present study suggest the validity of activating the abdominal muscle before ball contact during jump header shooting and provide useful insights for coaching jump header shooting. In this study, we focused on trunk muscle activity and clarified that abdominal muscle activity is important for jump header shooting. We think that the results of this study will provide coaching based on scientific evidence.

In contrast, we found that activity of the lower extremity muscles was greater during the push-off phase than during the other phases. Muscle activity of the lower extremity muscles may have increased in the push-off phase because the participants received a strong ground reaction force during take-off. Moreover, muscle activity in the lower extremities has been reported to decrease midair [20]. The results of the present study suggest that lower extremity muscle activity increases during the push-off phase when the participants’ feet receive ground reaction forces. Moreover, lower extremity muscle activity during the early and late floating phases decreases since the participant’s body floats midair.

In the trunk stabilization exercise group, ES muscle activity was significantly decreased post-intervention compared with that at pre-intervention. In the control group, there were no significant differences between muscle activity measured before and after intervention. The local muscle is located deep in the trunk and directly provides lumbar segmental stabilization [13]. Therefore, it is possible that the trunk stabilization exercises decreased the muscle activity of the ES because the segmental stability of the lumbar spine was improved. Furthermore, patients with chronic LBP demonstrate decreased activation of deep muscles and overactivation of superficial muscles such as the erector spinae [21]. Therefore, the decrease in ES muscle activity after trunk stabilization exercise suggests that the load on the back muscles may be reduced during jump header shooting. The training could be conducted before practice to reduce the load on the back muscles. However, since only one training session was performed, only the short-term training effects may have been obtained.

The strength of this study is that it revealed trunk muscle activity during jump header shooting in nineteen experienced soccer players. Experimental designs with experienced soccer players would be more difficult to recruit participants than studies with healthy students who have no exercise experience. In this study, it is significant that the measurements were conducted on experienced players. This study suggests that abdominal muscle activities are important for jump header shooting, and stabilization exercises may help reduce the load on the back muscle.

The present study has several limitations. First, though the target height of the soccer ball at the take-off point was standardized, the actual height reached by the ball was not analyzed. Trials that deviated significantly from the target ball arrival point were excluded. However, the detailed values were not calculated and might have deviated slightly from the target height. Second, we did not measure the speed of the throw ball. The same examiner threw the ball and maintained the ball speed. However, the ball speed was not calculated; thus, a small difference in the speed may have influenced the results. Finally, to compare the intervention effect, the experimental tasks were standardized. However, it should be considered that jump header shooting takes place in various situations. Moreover, regarding the examination of the comparison of intervention effects, it is possible that the number of participants in each group was rather small. The participants of this study were only healthy students. In the future, it is expected to clarify the effects of the intervention in participants with low back pain and to examine the long-term effects of the intervention.

## 5. Conclusions

During jump header shooting, the muscle activity of the IO increases prior to the increase in muscle activity of the RA and EO, and the muscle activity of all abdominal muscles increases immediately after take-off. The trunk stabilization exercise intervention decreased the muscle activity of the ES with immediate effect. Abdominal muscle activities are important for jump header shooting, and stabilization exercises may help reduce the load on the back muscle. These results provide useful insights for the coaching of jump header shooting.

## Figures and Tables

**Table 1:** Baseline characteristics of the trunk stabilization exercise group and control group.

	Exercise Group	Control Group	*p*-Value
Age (years)	20.2 ± 1.0	21.2 ± 1.0	0.037
Height (cm)	170.9 ± 6.4	174.7 ± 4.8	0.17
Weight (kg)	63.5 ± 9.2	67.5 ± 6.0	0.27
Soccer experience (years)	10.8 ± 1.4	9.8 ± 3.1	0.37

Group characteristics expressed as mean ± SD. Statistically significant difference: *p* < 0.05.

**Table 2:** Muscle activity in each group before and after intervention.

		Exercise Group		Control Group	
		Pre-Intervention	Post-Intervention	Pre-Intervention	Post-Intervention
RA	Push-off	27.4 ± 16.5	26.3 ± 23.3	37.7 ± 23.9	43.6 ± 24.1
	Early floating	63.9 ± 25.3	69.8 ± 35.8	71.4 ± 28.2	62.1 ± 32.2
	Late floating	29.1 ± 16.2	28.3 ± 16.4	23.0 ± 13.5	22.6 ± 14.7
IO	Push-off	76.1 ± 20.9	83.9 ± 23.0	94.9 ± 58.0	103.6 ± 63.3
	Early floating	98.5 ± 35.3	101.5 ± 40.9	96.3 ± 83.8	106.6 ± 98.9
	Late floating	60.3 ± 26.7	62.1 ± 22.5	69.2 ± 51.8	71.5 ± 53.6
EO	Push-off	35.1 ± 17.4	37.9 ± 23.1	39.1 ± 27.8	56.3 ± 49.6
	Early floating	73.9 ± 70.1	72.4 ± 74.1	107.1 ± 88.5	93.0 ± 69.0
	Late floating	30.4 ± 21.5	32.3 ± 26.5	26.0 ± 16.2	25.9 ± 15.9
ES	Push-off	19.9 ± 10.3	18.3 ± 9.6	19.8 ± 10.9	22.3 ± 10.9
	Early floating	19.7 ± 9.7	14.1 ± 12.3	27.7 ± 14.0	25.5 ± 9.7
	Late floating	29.1 ± 13.0	21.5 ± 7.0	24.5 ± 12.1	21.9 ± 11.3
GMa	Push-off	76.6 ± 25.3	69.8 ± 26.2	56.6 ± 31.3	60.6 ± 43.4
	Early floating	25.2 ± 16.7	21.4 ± 10.9	34.8 ± 19.6	35.3 ± 21.7
	Late floating	17.6 ± 6.8	19.9 ± 9.4	27.4 ± 11.6	28.4 ± 14.3
BF	Push-off	36.9 ± 18.7	41.0 ± 18.7	37.4 ± 27.8	30.1 ± 20.3
	Early floating	22.8 ± 14.2	20.0 ± 11.7	27.3 ± 24.4	24.1 ± 23.4
	Late floating	26.2 ± 15.3	22.6 ± 15.1	24.7 ± 15.0	22.9 ± 13.8
RF	Push-off	102.4 ± 33.8	110.8 ± 32.3	117.0 ± 47.8	110.3 ± 42.4
	Early floating	59.4 ± 26.1	55.6 ± 27.6	70.8 ± 37.0	72.1 ± 34.9
	Late floating	18.2 ± 8.7	16.6 ± 7.1	24.9 ± 15.6	24.8 ± 15.6
MG	Push-off	132.1 ± 42.4	127.0 ± 43.1	120.3 ± 34.9	114.4 ± 37.0
	Early floating	34.3 ± 16.4	31.9 ± 14.1	30.7 ± 14.7	31.1 ± 21.4
	Late floating	52.2 ± 8.6	57.3 ± 13.6	56.7 ± 11.0	59.9 ± 18.8

Muscle activity data expressed as mean ± SD. Abbreviations: left rectus abdominis (RA), internal oblique (IO), external oblique (EO), erector spinae (ES), gluteus maximus (GMa), biceps femoris (BF), rectus femoris (RF), gastrocnemius medial head (MG), Unit of measurement: normalized EMG activity, expressed as % of maximum voluntary contraction (MVC).

**Table 3:** Hang time in each group before and after intervention.

	Hang Time Before Intervention (s)	Hang Time After Intervention (s)	*p*-Value
Exercise group	0.43 ± 0.06	0.42 ± 0.07	0.625
Control group	0.45 ± 0.06	0.44 ± 0.04	0.364

Group characteristics expressed as mean ± SD. Statistically significant difference: *p* < 0.05.

## Data Availability

The data that support the findings of this study are available from the corresponding author.
